# Executive Cognitive Dysfunction and ADHD in Cocaine Dependence: Searching for a Common Cognitive Endophenotype for Addictive Disorders

**DOI:** 10.3389/fpsyt.2013.00126

**Published:** 2013-10-21

**Authors:** Paulo Jannuzzi Cunha, Priscila Dib Gonçalves, Mariella Ometto, Bernardo dos Santos, Sergio Nicastri, Geraldo F. Busatto, Arthur Guerra de Andrade

**Affiliations:** ^1^Laboratory of Psychiatric Neuroimaging (LIM-21), Department of Psychiatry, Faculty of Medicine, University of São Paulo (USP), São Paulo, SP, Brazil; ^2^Center for Interdisciplinary Research on Applied Neurosciences (NAPNA), USP, São Paulo, SP, Brazil; ^3^Interdisciplinary Group of Studies on Alcohol and Drugs (GREA), Faculty of Medicine, USP, São Paulo, SP, Brazil; ^4^Equilibrium Program, Department of Psychiatry, Faculty of Medicine, USP, São Paulo, SP, Brazil; ^5^School of Nursing, USP, São Paulo, SP, Brazil

**Keywords:** executive dysfunction, ADHD, cocaine, addiction, impulsivity, prefrontal cortex

## Abstract

**Background:** Cocaine-dependent individuals (CDI) present executive cognitive function (ECF) deficits, but the impact of psychiatric comorbidities such as Attention-Deficit Hyperactivity Disorder (ADHD) on neuropsychological functioning is still poorly understood. The aim of this study was to investigate if CDI with ADHD (CDI + ADHD) would have a distinct pattern of executive functioning when compared with CDI without ADHD (CDI).

**Methods:** We evaluated 101 adults, including 69 cocaine-dependent subjects (divided in CDI and CDI + ADHD) and 32 controls. ECF domains were assessed with Digits Forward (DF), Digits Backward (DB), Stroop Color Word Test (SCWT), the Wisconsin Card Sorting Test (WCST), and the Frontal Assessment Battery (FAB). DSM-IV criteria for ADHD were used for diagnosis and previous ADHD symptoms (in the childhood) were retrospectively assessed by the Wender-Utah Rating Scale (WURS).

**Results:** There were no significant differences between CDI + ADHD, CDI, and controls in estimated intellectual quotient (IQ), socioeconomic background, education (in years), and pre-morbid IQ (*p* > 0.05). SCWT and WCST scores did not differ across groups (*p* > 0.05). Nevertheless, CDI and CDI + ADHD performed more poorly than controls in total score of the FAB (*p* < 0.05). Also, CDI + ADHD did worse than CDI on DF (*F* = 4.756, *p* = 0.011), DB (*F* = 8.037, *p* = 0.001), Conceptualization/FAB (*F* = 4.635, *p* = 0.012), and Mental flexibility/FAB (*F* = 3.678, *p* = 0.029). We did not find correlations between cocaine-use variables and neuropsychological functioning, but previous ADHD symptoms assessed by WURS were negatively associated with DF (*p* = 0.016) and with the total score of the FAB (*p* = 0.017).

**Conclusion:** CDI + ADHD presented more pronounced executive alterations than CDI and CDI exhibited poorer cognitive functioning than controls. Pre-existing ADHD symptoms may have a significant negative impact on executive dysfunction in CDI. It remains to be investigated by future studies if symptoms such as impulsivity or a pre-existing ECF dysfunction could represent underlying cognitive endophenotypes that would substantially increase the risk for acquiring addictive disorders.

## Introduction

There is accumulating evidence from cognitive neuroscience research that cocaine-dependent individuals (CDI) present prefrontal cortex (PFC) functional and structural abnormalities (1–5), which are associated with executive cognitive function (ECF) deficits (6–8). Data suggest that regional metabolic alterations seen in CDI are associated with changes in brain dopamine activity (2, 9, 10) as well as with neuronal injury in the frontal cortex in both frontal gray and white matter (5, 11–13). There are also several studies showing that chronic cocaine-use is associated with attention, memory, and executive impairments (7, 14–19).

Our research group has shown that cocaine-use is associated with hypoperfusion in several areas of the brain (3) and that CDI present several cognitive impairments which have real-life implications (16–18). We have recently evaluated 30 CDI, after 2 weeks of abstinence, and compared with 32 healthy individuals in frontal executive tasks. CDI performed more poorly than controls in digits forward (DF), digits backward (DB), and on the Frontal Assessment Battery (FAB) (17). The FAB is a brief neuropsychological battery devised by neurologists, composed of six subtests which evaluate different ECF-related functions (20). The performance on the six subtests of the FAB gives a global score suggesting a “descriptive pattern of ECF in a given patient” (20). In our study, CDI were cognitively impaired in 50% (3/6) of the cognitive domains assessed by the FAB: abstract reasoning, motor planning, and cognitive flexibility. The results on the FAB were correlated with traditional frontal/executive tasks, showing that the FAB is a good indicator of severity of the executive dysfunction in CDI. Another recent study from our group aimed to investigate a possible link between ECF-related tasks and what happens in the real social life of CDI. The results indicated that impaired ECF in CDI was correlated with their higher levels of social dysfunction, in several areas of the social domain, including work, leisure, family, and finances (18). The relevance of executive dysfunction in CDI is also justified because ECF-related impairments are associated with poor treatment retention in cognitive-behavioral therapy (CBT) (21, 22) and with relapses (23).

However, it is difficult to determine to what extent the substance *per se* leads to ECF alterations through its influence on brain functioning, or if the ECF deficits would represent pre-morbid factors which may lead to stimulant dependence (5) and to more intense neuropsychological deficits. According to recent studies, ECF impairments may be both predisposing factors and/or negative consequences of CDI (17, 24–27). Fillmore and Rush (27) have argued that a common cognitive characteristic of CDI is an executive deficit, which makes them more vulnerable to risky behaviors and substance abuse. Animal studies also reinforce the hypothesis that a impaired self-control, impulsivity, and ECF alterations may be candidates neurocognitive endophenotypes that predates the emergence of addictions (28–30); those symptoms are very common across disorders such as Attention-Deficit Hyperactivity Disorder (ADHD) (5, 30).

Attention-deficit hyperactivity disorder is characterized as a neuropsychiatric disorder, with inattention, hyperactivity, and impulsivity symptoms (31). ADHD is significantly more prevalent among CDI than in the general population (32). The high prevalence of ADHD among CDI could be a significant confounding factor for neuropsychological studies, as ADHD *per se* is associated with neuropsychological deficits that affect predominantly ECF and also interfere in daily life activities (33–35). The ADHD cognitive endophenotype includes deficits in response inhibition that leads to impulsivity (30, 36), and this is considered a possible vulnerability marker for cocaine dependence (27). Moreover, it has been suggested that measures of response inhibition can help identify genetic susceptibility to ADHD (36).

However, there is not enough evidence to establish if the ECF deficits are pre-existing factors in CDI, which could lead to real-life problems and then make the subject more vulnerable to drug addiction, or if the cocaine-use *per se* would be the main factor that impairs ECF leading to the persistence of cocaine-use despite negative consequences (18). We consider that there is still inconsistency regarding the findings of attention and executive deficits among CDI, due to different methodologies applied and to the lack of more controlled studies (37). Further studies are necessary to verify the nature and severity of the attention and executive deficits associated with cocaine-use, as they have a direct clinical implication in treatment (6, 7, 16, 32). To date, few studies have investigated the role of ADHD in cocaine dependence. One study showed that although cocaine abusers performed significantly more poorly than controls on several neuropsychological tasks, there was no significant relationship between measures of childhood ADHD symptoms and neuropsychological performance (38). On the other hand, more recently, it was observed that ADHD symptoms were important modulators of cognitive function in CDI, suggesting that cocaine-use and ADHD symptoms seem to have mutual aggravating effects in executive impairments (19).

Considering that there are still controversies about the role of ADHD diagnosis in cognitive performance of CDI, our aim was to evaluate the ECF deficits among CDI and to investigate the impact of an ADHD diagnosis on these patients. Our hypothesis was that CDI with ADHD diagnosis would present more pronounced deficits on ECF and that ADHD symptoms and cocaine-use-related variables such as age at onset would be correlated with the degree of ECF deficits in these patients.

## Materials and Methods

### Participants

Hundred and one subjects participated in this study. All the CDI (with or without ADHD, *n* = 69) met the DSM-IV-TR criteria (31) for cocaine dependence at the time of admission to the treatment program. The CDI were recruited from two inpatient units: (1) the Interdisciplinary Group of Studies on Alcohol and Drugs (GREA) at the University of São Paulo (USP); (2) the Association for the Promotion of Prayer and Work (APOT) in Campinas (SP, Brazil). Exclusion criteria included were: (1) past or current major DSM diagnosis of psychotic disorders, or a current diagnosis of bipolar disorders; (2) met DSM criteria for opioid dependence; (3) had a history of neurological condition such as epilepsy and/or head injuries with loss of consciousness for longer than 30 min, strokes and intracranial hemorrhages; (4) had prior diagnosis of learning disorder; (5) had intellectual quotient (IQ) less than 70. The CDI were all treatment-seeking cocaine-dependent patients evaluated after at least 1 week of abstinence. The abstinence was verified by self-report and supervised by the clinical staff of the inpatient units. For the majority of these patients (*n* = 39, 56.5%), two urine tests were used to verify recent cocaine-use (if positive) and after to verify their abstinence (if negative), since recent cocaine-use may mask cognitive impairments in CDI (39). The neurocognitive performance of the CDI was compared to a control group which consisted of 32 healthy individuals, who were volunteers, recruited in the city of São Paulo. The control group consisted of employes from the public hospital where the research center (GREA) is located (*n* = 2) and the local police department (*n* = 12). We also recruited adult students from a public school (*n* = 18). The transport costs of the volunteers were reimbursed. The exclusion criteria for the control group were: (1) met DSM criteria for any psychoactive substance dependence other than nicotine; (2) the same exclusion criteria of the cocaine group.

### Procedures and ethical considerations

The data presented in this report were collected between January 2001 and March 2013. The research protocol satisfied the Helsinki Declaration and was approved by the University of Sao Paulo Review Board (CAPPesq). After signing an informed consent, participants were interviewed by either a clinical psychologist or a psychiatrist. The interview questions covered demographics, drug use, and the consequences of drug use on their psychosocial functioning. Investigators obtained initial demographic and clinical information by a semi-structured interview used by neuropsychologists at our research center (17). The semi-structured neuropsychological interview included basic information such as name, address, gender, age, ethnic, handedness, educational background, professional activities, socioeconomic level, as well as questions about the medical past and current history, neurodevelopmental history, and neurocognitive complaints associated with cocaine and drug use (16, 18).

### Mood, anxiety, and drug use evaluation

Psychological and psychiatric symptoms were assessed by the Beck Depression Inventory (BDI) (40) and the State-Trait Anxiety Inventory (STAI) (41). Alcohol, tobacco, and other drug use, as well as the consequences of drug use among the cocaine group, were assessed using the Cocaine Addiction Severity Test (CAST) and Cocaine Assessment Profile (42) or the ASI-6 *Addiction Severity Index* (43).

### Neuropsychological measures

#### Digits forward and backward, from the revised version of the Wechsler Adult Intelligence Scale

Digits forward was used was used to measure attention span and DB working memory (44). In DF the examinee needs to repeat a sequence of random numbers in the same order and DB in the reverse order (45).

#### Stroop color word test

It was designed to measure selective attention, cognitive flexibility, and inhibitory control (45, 46). Here it was used a Stroop color word test (SCWT) Portuguese version, published elsewhere (17).

#### Wisconsin card sorting test

The 64 card version (47, 48), translated and validated for use in Brazil (17, 49). This task evaluates ECF such as: mental flexibility, abstract reasoning, capacity to maintain the cognitive setting and self-monitoring (45).

#### Frontal assessment battery

The administration of the FAB takes approximately 10 min; each subtest is scored from 0 (minimum score) to 3 (maximum score) and the total score of the FAB is the sum of the scores in the six subtests (the FAB’s total score ranges from 0 to 18) (20). The FAB was translated into Portuguese (17, 50) and detailed information about instructions of the FAB are described elsewhere (20). The six subtests are: conceptualization, mental flexibility, motor programing, sensitivity to interference, inhibitory control, and environmental autonomy.

#### Intellectual functioning

We used short forms of the Wechsler Intelligence Scale for Adults – Revised [WAIS-R, (44)] and the Wechsler Adult Scale of Intelligence (WASI) to evaluate an estimated IQ which is a reliable measure of Full Scale IQ.

### Objective measurement of ADHD symptoms and diagnosis

#### Wender-Utah rating scale

It evaluates retrospectively different symptoms of ADHD. Here we used an abbreviated version of the Wender-Utah Rating Scale (WURS) comprising 25 of the 61 items from the original scale, which has shown to better discriminate between patients with ADHD and a non-patient comparison group (51). Participants were asked to self-report if they had experienced those ADHD symptoms during the childhood (until the age of 12 years old) on a Likert-type scale scored 0 (not at all or very slightly), 1 (mildly), 2 (moderately), 3 (quite a bit), and 4 (very much). The WURS minimum score is 0 and the maximum score is 100. The total was calculated and participants were considered as having preexistent ADHD significant symptoms if they scored 46 or above, which has shown to be a reliable estimate for ADHD diagnosis in the childhood. More specifically, in a validation study, a cutoff score of 46 or higher correctly identified 86% of the patients with ADHD and 99% of the normal subjects (51).

#### ADHD diagnosis by DSM-IV-TR

It is based in nine symptoms of Inattention and nine symptoms of Hyperactivity/Impulsivity (31). The patient must fulfill at least six of nine (six or more) symptoms in each group or in both groups (Inattention and Hyperactivity/Impulsivity). The symptoms must have persisted for at least 6 months to a degree that is inconsistent with developmental level and that impact directly on social and academic/occupational activities. A requirement for the diagnosis of ADHD in adults is a childhood history of ADHD which was measured by WURS (right above).

#### Definition of CDI + ADHD, CDI, and controls

We only considered subjects in the group called CDI + ADHD if they had fulfilled both confirmation of the adult ADHD diagnosis by DSM-IV-TR criteria (31) and if they had a WURS total score of 46 or higher (51).

### Statistical analyses

Differences in performance on neuropsychological tests were assessed with unpaired *t*-tests and categorical variables were evaluated using Fisher’s exact test. The normal distribution of each cognitive variable was confirmed by the Kolmogorov–Smirnov test. The level of statistical significance was α = 0.05 and all statistical tests were two tailed. Comparative analysis considering three groups (CDI, CDI + ADHD, and controls) was made using Analysis of Variance (ANOVA) and Bonferroni *post hoc* testing. Additionally, between-group analysis of cognitive functioning was conducted using Analysis of Covariance (ANCOVA) with age and gender as covariates. Correlation between neuropsychological measures [i.e., DF, DB, SCWT, Wisconsin Card Sorting Test (WCST), and FAB], cocaine-use variables (i.e., age at onset, abstinence, and duration of cocaine-use), and WURS (total score) was assessed by the Spearman correlation coefficient (*r*_s_). All statistical analyses were conducted using Statistical Package for the Social Science (SPSS) software version 14.0 for Windows.

## Results

### Demographic and clinical characteristics

Socio-demographic and some clinical characteristics of the groups are described in Table 1.

**Table 1:** **Socio-demographic variables, intellectual functioning, and substance use of the CDI, CDI ***+*** ADHD, and healthy controls**.

	CDI (*n* = 58)	CDI + ADHD (*n* = 11)	Controls (*n* = 32)	*p*
Age	31.24 (±7.39)	28.64 (±8.27)	26.75 (±5.55)	0.015*
Gender (male/female)	56/2	8/3	32/0	0.001*
Education (years)	11.78 (±3.41)	10.82 (±3.60)	10.78 (±2.20)	0.292
Socioeconomic level
A	10.3%	18.2%	3.1%	0.295
B	48.3%	45.5%	34.4%	
C	32.8%	36.4%	43.8%	
D	8.6%	0%	18.8%	
Ethnicity (Brazilian White/African Brazilian)	18/40	4/7	27/5	0.000*
Handedness (right handed/left-handed/ambidextrous)	52/0/6	10/0/1	26/5/1	0.015*
Estimated intellectual quotient (IQ)	96.36 (±14.11)	93.91 (±16.66)	101.06 (±12.82)	0.210
Vocabulary (WAIS-R or WASI)	49.55 (±12.25)	47.73 (±12.56)	47.31 (±8.79)	0.642
WURS	24.91 (±16.23)	61.73 (±12.58)	13.77 (±14.09)	0.000*

CDI, cocaine-dependent individuals; CDI + ADHD, cocaine-dependent individuals with attention deficit/hyperactivity disorder; SD, Standard Deviation (±); WAIS-R, Wechsler Adult Intelligence Scale-Revised; WASI, Wechsler Abbreviated Scale of Intelligence; WURS, Wender-Utah Rating Scale; Quantitative variables were compared using ANOVA and categorical variables were compared using Pearson Chi-Square;*p < 0.05 – statistically significant. Socioeconomic levels were classified according to Brazilian norms (ABIPEME): A = highest socioeconomic level and D = lowest socioeconomic level.

The three groups did not differ in educational level (in years), socioeconomic level, estimated IQ, and vocabulary score. However we found differences among groups regarding age, gender, ethnicity, handedness, and childhood ADHD scores (WURS). Controls were younger, male, more frequently white, and left-handed, in comparison to both CDI + ADHD (*n* = 11) and CDI (*n* = 58). CDI + ADHD had significantly higher WURS scores, followed by CDI and normal controls (Table 1).

### Cocaine, alcohol, and other substance recent use by the CDI and CDI + ADHD

Groups with a history of cocaine dependence (CDI and CDI + ADHD) did not differ significantly in terms of the main cocaine-use variables, such as age at onset, duration (in years), and length of abstinence (Table 2). They also showed a similar pattern of recent use for other substances, such as alcohol, cannabis, tobacco, heroin, amphetamine, sedatives, and LSD. Differences were only detected for alcohol and tobacco recent use when comparing both groups of patients (CDI and CDI = ADHD) with controls. There were no differences between the percentages of cannabis and multiple substance abusers among the groups of patients.

**Table 2:** **Cocaine, alcohol, and other drug use in the CDI and CDI ***+*** ADHD**.

	CDI (*n* = 58)	CDI + ADHD (*n* = 11)	Controls (*n* = 32)	*p*
**COCAINE-USE**				
Age at onset	18.81 ± 4.57	18.27 ± 6.42	–	0.739
Duration (years)	10.71 ± 6.98	8.27 ± 4.38	–	0.270
Abstinence (days)	12.40 ± 9.16	12.09 ± 4.30	–	0.914
**ALCOHOL AND OTHER SUBSTANCES (RECENT USE, *n*)**				
Alcohol	53.4%	45.5%	9.4%	0.000
Tobacco	69%	81.8%	12.5%	0.000
Cannabis	39.7%	54.5%	–	0.278
Heroin	1.7%	0%	–	1.000
Amphetamine	1.7%	0%	–	1.000
Sedatives	20.7%	27.3%	–	0.694
Multiple substance users	27.3%	27.6%	–	1.000

CDI, cocaine-dependent individuals; CDI + ADHD, cocaine-dependent individuals with attention deficit/hyperactivity disorder; Recent use = was defined as substance use for at least three times a week in the last 30 days; Quantitative variables (about cocaine-use) were compared using Student *t*-test for comparisons among CDI and CDI + ADHD; Categorical variables (about alcohol and other substances) were compared using Pearson Chi-Square and Fisher’s Exact Test; *p < 0.05 – statistically significant; *p* refers to comparative analyses only between CDI and CDI + ADHD except for alcohol and tobacco recent use (CDI × CDI + ADHD × Controls).

### Neuropsychological findings

The ANOVA including the three groups (CDI, CDI + ADHD, and controls) did not show any statistically significant difference among patients and controls in all measures of SCWT and WCST. However, groups differed in attention (DF: *F* = 4.756, *p* = 0.011) and in working memory (DB: *F* = 8.037, *p* = 0.001) tasks (Table 3). *Post hoc* comparisons showed that the difference in DF was due to worse performance of CDI + ADHD in comparison to both CDI (*p* = 0.013) and controls (*p* = 0.011). CDI did not differ significantly from controls in this task (DF). Differences in the test of working memory (DB) were due to a lowered performance of both groups of cocaine-dependents (CDI + ADHD and CDI) when directly and separately compared with controls (CDI + ADHD × Controls, *p* = 0.008; CDI × Controls, *p* = 0.001).

**Table 3:** **Performance of CDI, CDI ***+*** ADHD, and healthy controls in traditional neuropsychological tests designed to evaluate executive cognitive functioning (ECF)**.

Neurocognitive functions	Neuropsychological tests		CDI mean (±SD) (*n* = 58)	CDI + ADHD mean (±SD) (*n* = 11)	Controls mean (±SD) (*n* = 32)	*p*
Attention and executive functioning	SCWT	Part I (sec)	14.460 ± 3.63	13.745 ± 2.31	14.58 ± 4.22	0.81
		Part II (sec)	17.16 ± 4.21	18.63 ± 4.41	17.29 ± 4.71	0.59
		Part III (sec)	26.68 ± 8.68	28.86 ± 9.12	26.08 ± 8.88	0.66
	WCST (64-cards)	Errors	19.22 ± 9.64	24 ± 11.16	19.78 ± 9.10	0.32
		Perseverative errors	9.79 ± 6.18	12.91 ± 7.71	10.22 ± 5.57	0.31
		Failure to maintain set	0.71 ± 0.84	0.27 ± 0.47	0.63 ± 0.79	0.25
		Categories*	2.83 ± 1.27	2.36 ± 1.80	2.72 ± 1.30	0.57
	Digits (WAIS-R)	Forward (DF)	6.45 ± 1.97	4.36 ± 2.33	6.63 ± 2.51	0.01*
		Backward (DB)	4.78 ± 1.58	4.27 ± 1.49	6.25 ± 2.36	0.00*

CDI, cocaine-dependent individuals; CDI + ADHD, cocaine-dependent individuals with attention deficit/hyperactivity disorder; SD, Standard Deviation [±]; SCWT, Stroop Color Word Test; sec, seconds; WCST, Wisconsin Card Sorting Test; WAIS-R, Wechsler Adult Intelligence Scale-Revised; *p < 0.05 – statistically significant; *p* values were calculated using ANOVA.

**Figure 1:**
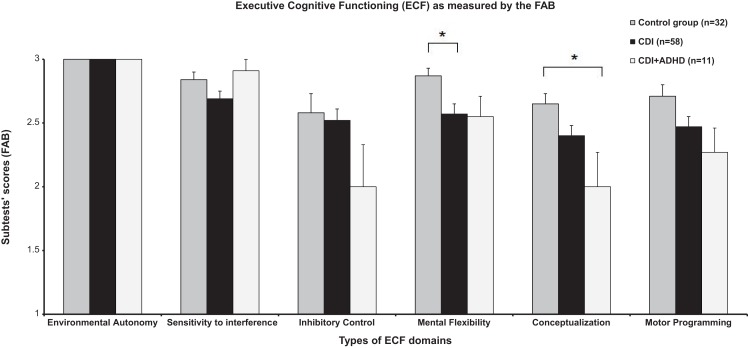
**Neurocognitive performance of CDI, CDI + ADHD and healthy controls in the six cognitive domains of the Frontal Assessment Battery (FAB)**. CDI, cocaine-dependent individuals; ADHD, attention-deficit hyperactivity disorder; FAB, Frontal Assessment Battery; ECF, executive cognitive functioning; [*] indicate ECF domains significantly different between groups by ANOVA (*p* < 0.05); vertical lines represent standard errors SE (±).

We also found statistically significant differences in FAB’s Conceptualization (*F* = 4.635, *p* = 0.012), FAB’s Mental flexibility (*F* = 3.678, *p* = 0.029) and in the total score of the FAB (*F* = 7.992, *p* = 0.001). *Post hoc* analyses showed that the significant difference in Conceptualization was due to a worse performance of CDI + ADHD when directly compared with the normal controls (*p* = 0.010). Difference detected in Mental Flexibility by ANOVA was associated with a lowered performance of CDI compared to controls (*p* = 0.029, Figure 1). Finally, differences among groups in the total’s FAB’s score were related to a worse performance of both group of patients (CDI and CDI + ADHD) when directly and separately compared with controls (CDI + ADHD × Controls, *p* = 0.001; CDI × Controls, *p* = 0.010).

### Correlations of cocaine-use variables and childhood ADHD scores by the WURS with measures of executive functioning

Associative analyses were conducted in order to investigate the influence of the following variables associated with cocaine-use and childhood ADHD symptoms on executive functioning: age at onset of cocaine-use, duration of cocaine-use (in years), length of abstinence from cocaine (in days), and WURS total scores.

Variables regarding cocaine-use were not correlated with executive functioning in this study. We found a significant negative association between childhood ADHD symptoms as measured by WURS and DF (*r*_s_ = −0.286; *p* = 0.016) and WURS with the FAB total score (*r*_s_ = −0.285; *p* = 0.017). Correlations were analyzed considering both groups of patients together (CDI and CDI + ADHD) (Table 4).

**Table 4:** **Correlation between drug use variables, childhood ADHD, and neuropsychological measures among the CDI and CDI + ADHD**.

Correlation (*r*_s_)	Cocaine age at onset (years)	Cocaine lifetime use (years)	Cocaine abstinence length (days)	Childhood ADHD (WURS)
**NEUROPSYCHOLOGICAL MEASURES**				
FAB’s total score	0.016	0.015	−0.078	−0.285*
DF(WAIS-R)	0.138	0.015	−0.001	−0.286*
DB (WAIS-R)	0.045	−0.215	0.145	−0.014

CDI, cocaine-dependent individuals; CDI + ADHD, cocaine-dependent individuals with attention deficit/hyperactivity disorder; *r*_s_, Spearman correlation coefficient; FAB, Frontal Assessment Battery; DF, digits forward; DB, digits backward; WURS, Wender-Utah Rating Scale; correlations were considered statistically significant if *p < 0.05.

Additionally, considering the differences between samples, between-group analysis of cognitive functioning was conducted using Analysis of Covariance (ANCOVA) with age and gender as covariates, and the results described above remained significant (*p* < 0.05).

## Discussion

In this study, we evaluated CDI with and without a diagnosis of ADHD, and compared them with healthy individuals. Our data confirmed our previous studies (16, 17) indicating a poor executive functioning in CDI when compared with normal controls, especially in a brief neuropsychological battery devised for evaluation of prefrontal functioning (FAB) (17). Moreover, a previous diagnosis of ADHD among CDI (CDI + ADHD) was associated with a more pronounced negative impact on executive dysfunction, especially in tasks requiring attentional spam and abstraction ability (concept formation). Our correlation analysis showed consistency of these findings, since the self-reported presence of childhood symptoms of ADHD was negatively associated with actual measures of attention and executive functions. Our findings confirm that ADHD symptoms have aggravating effects in executive impairments in CDI and also suggest that the pre-existence of executive deficits (suggested by self-reported ADHD symptoms in the childhood) may represent risk factors for a more intense degree of cognitive impairment. Also, even CDI without ADHD presented significant executive impairments, when compared with controls, so cocaine *per se* may negatively interfere in brain functioning and executive functioning.

The ECF deficits observed in CDI, in general, could support the lack of self-control and persistence of compulsive behavior regarding cocaine-use, despite of the adverse consequences on health and social functioning (18, 52, 53). In other words, the inability to change a previous behavior (mental flexibility deficits) and the difficulty to acquire and maintain a healthier life-style (conceptualization and working memory deficits) might lead to social dysfunction, treatment drop-outs, and relapses, which are often observed in this population (18, 21–23).

Our findings support the idea that ADHD-related executive dysfunction may represent a predisposing factor, which is brought about by either genetic factors, or early environmental factors (e.g., emotional stress), which may negatively impact the normal development of the PFC, which in turn leads to poor decision-making, which heightens the risk for acquiring addictive disorders (18). The literature is consistent with the hypothesis that ADHD play a powerful role in increasing the likelihood of cocaine and other substance abuse. ADHD symptoms could represent a critical risk factor for cocaine-induced changes of the dopaminergic system and brain alterations (54). The intermittent dopamine stimulation that follows cocaine-use produces acute changes in the synapses and in dopaminergic neurons in the PFC via the striato-thalamo-frontal circuit associated with pleasure and reward (55). In the long-term, cocaine-use may cause structural and functional impairments in certain brain areas, including the PFC (4, 5, 52, 53), representing an additional negative impact on PFC functioning in these subjects (5, 18, 56).

In other words, while ADHD symptoms since the childhood among cocaine abusers have been considered confounding factors for the neuropsychological evaluation, since ADHD *per se* is associated to neuropsychological deficits affecting predominantly ECF, ADHD-related symptoms may be an explaining suggestive factor for the future development of cocaine dependence. Much more than a “confounding factor,” ADHD might be an “explaining factor” for the understanding of possible cognitive endophenotypes associated with cocaine dependence. It is possible that CDI + ADHD would represent a subgroup of patients whose cocaine-use may be associated with a certain type of “self-treatment” for pre-existing ADHD symptoms (32). Also, it is possible that another “subgroup” of CDI would present subclinical ADHD symptoms (as they did not achieve the cutoff score in WURS for ADHD, on the other side symptoms of ADHD in CDI were two times more intense when compared with the control group, *p* = 0.005), to which similar pharmacological and behavioral strategies used for ADHD could be beneficial. For example, a recent study showed that oral methylphenidate (MPH, which is a traditional pharmacological treatment for ADHD) may induce better performance (reducing errors) on the SCWT leading to a more careful responding by reducing brain activity in the dorsolateral PFC of CDI as measured by fMRI (57). The authors argued that MPH modulates dopamine in the PFC for both CDI and controls, leading to a better error-related processing, with an effect that is more significant in CDI (57). For CDI + ADHD, the use of oral MPH could be very beneficial, considering that MPH-elicited dopamine increases may improve ADHD symptoms and ventral striatum functioning in adults with ADHD (58). In addition, it could be also relevant to measure the role of other pharmacological treatments, such as mood stabilizers to treat impulsivity and risky behaviors in CDI + ADHD, CDI, and in other addicted patients (59–61). The use of an indicated pharmacological treatment for them could be beneficial, in order to help them to stop cocaine-use that may worsen a previous existent executive function deficit. The abuse potential of each pharmacological treatment, however, is an issue that should always be taken into account (61). Also, early detection and treatment of ADHD in children seems to reduce the risk for a later substance-use disorder (SUD) (62).

Other studies have suggested ECF deficits as possible cognitive endophenotypes to addictive disorders (29, 63–65). Healthy adults with family history of alcohol dependence presented more ECF deficits, impulsivity traits (64), and also abnormal brain response during a verbal working memory task (63) than individuals without alcoholics in their families. A similar result was observed in unaffected siblings of stimulants (cocaine and amphetamine) dependents, which exhibited significantly lower ECF and response control than healthy controls (66). After review the findings from high-risk research, problem gamblers, and genetic association studies, Verdejo-García et al. (67) concluded that impulsivity is a pre-existing vulnerability marker for SUDs. In fact, not only impulsivity, but also risk-taking and stress responsivity (68) could be cognitive and behavioral traits guided by genetic factors, constructed under certain environmental influences, which could represent relevant factors on the vulnerability to addictive disorders.

However, some limitations of this study need to be highlighted. First, the present study included a small sample of CDI + ADHD, but we have to bear in mind that our rigidity on criteria for the diagnosis of ADHD was more appropriated to direct our analysis. Second, since the present data are cross-sectional, it was not possible to determine whether self-reported ADHD childhood symptoms detected by WURS indeed afford a reliable method for measuring ECF impairments in the childhood. On the other hand, recent studies have shown that retrospective information provided by adults with ADHD has agreement with parents’ reports (69). Also, our correlation analysis indicated a direct link between previous ADHD symptoms and the actual executive dysfunction, suggesting that our data are in consonance with findings that suggest an aggravating effect of ADHD symptoms on cognitive dysfunction (19), but further prospective studies should better investigate our hypothesis. Third, we evaluated samples with different ages and genders, but the results remained significant when including age and gender as covariates in all analysis. Fourth, alcohol dependence was not an exclusion criteria and we have previously suggested that alcohol use may be associated with frontal lobes impairments (50), but it is unlikely that this may have biased our results because we found a similar proportion of patients with recent alcohol use in CDI and CDI + ADHD. We believe that addicted patients independent of the used drug could have different severities of frontal lobe functions, different ADHD disturbances and different temperaments, so they need specific treatment aims and different treatment strategies. Fifth, it was not possible to determine the reversibility of executive deficits, because the abstinence period in our sample was too short, so future studies should investigate if executive dysfunction in CDI + ADHD and CDI could be reversible during cocaine prolonged abstinence.

In summary, our data confirms that CDI + ADHD have more pronounced neuropsychological alterations than CDI and controls. Correlation analysis suggested that a possible pre-existing executive dysfunction in CDI + ADHD could lead to a more salient pattern of cognitive impairment and that it is a relevant candidate to a possible cognitive endophenotype of cocaine dependence that influences on later neuropsychological functioning. It remains to be investigated by prospective studies if pre-morbid ECF-related alterations would represent commonalities among the other substance or behavioral addictive disorders (i.e., gambling, sexual compulsive behavior, among others) as well as their genetic and neuroimaging underpinnings.

## Conflict of Interest Statement

The authors declare that the research was conducted in the absence of any commercial or financial relationships that could be construed as a potential conflict of interest.

## References

[1] Volkow ND Mullani N Gould KL Adler S Krajewski K Cerebral blood flow in chronic cocaine users: a study with positron emission tomography. Br J Psychiatry (1988) 152:641–8 10.1192/bjp.152.5.641 3262397

[2] Volkow ND Fowler JS Wolf AP Hitzemann R Dewey S Bendriem B Changes in brain glucose metabolism in cocaine dependence and withdrawal. Am J Psychiatry (1991) 148(5):621–6 2018164 10.1176/ajp.148.5.621

[3] Nicastri S Buchpiguel CA Andrade AG Anormalidades de fluxo sangüíneo cerebral em indivíduos dependentes de cocaína. Rev Bras Psiquiatr (2000) 22(2):42–50 10.1590/S1516-44462000000200003

[4] Goldstein RZ Alia-Klein N Tomasi D Zhang L Cottone LA Maloney T Is decreased prefrontal cortical sensitivity to monetary reward associated with impaired motivation and self-control in cocaine addiction? Am J Psychiatry (2007) 164(1):43–51 10.1176/appi.ajp.164.1.43 17202543 PMC2435056

[5] Ersche KD Williams GB Robbins TW Bullmore ET Meta-analysis of structural brain abnormalities associated with stimulant drug dependence and neuroimaging of addiction vulnerability and resilience. Curr Opin Neurobiol (2013) 23(4):615–24 10.1016/j.conb.2013.02.017 23523373

[6] Strickland TL Mena I Villanueva-Meyer J Miller BL Cummings J Mehringer CM Cerebral perfusion and neuropsychological consequences of chronic cocaine use. J Neuropsychiatry Clin Neurosci (1993) 5(4):419–27 8286941 10.1176/jnp.5.4.419

[7] Gottschalk C Beauvais J Hart R Kosten T Cognitive function and cerebral perfusion during cocaine abstinence. Am J Psychiatry (2001) 158(4):540–5 10.1176/appi.ajp.158.4.540 11282686

[8] Tomasi D Goldstein RZ Telang F Maloney T Alia-Klein N Caparelli EC Widespread disruption in brain activation patterns to a working memory task during cocaine abstinence. Brain Res (2007) 1171:83–92 10.1016/j.brainres.2007.06.102 17765877 PMC2048813

[9] Kalivas PW Glutamate systems in cocaine addiction. Curr Opin Pharmacol (2004) 4(1):23–9 10.1016/j.coph.2003.11.002 15018835

[10] Volkow ND Fowler JS Wang GJ Baler R Telang F Imaging dopamine’s role in drug abuse and addiction. Neuropharmacology (2009) 56(Suppl 1):3–8 10.1016/j.neuropharm.2008.05.022 18617195 PMC2696819

[11] Chang L Ernst T Strickland T Mehringer CM Gender effects on persistent cerebral metabolite changes in the frontal lobes of abstinent cocaine users. Am J Psychiatry (1999) 156(5):716–22 10327904 10.1176/ajp.156.5.716

[12] Matochik JA London ED Eldreth DA Cadet JL Bolla KI Frontal cortical tissue composition in abstinent cocaine abusers: a magnetic resonance imaging study. Neuroimage (2003) 19(3):1095–102 10.1016/S1053-8119(03)00244-1 12880835

[13] Moreno-López L Catena A Fernández-Serrano MJ Delgado-Rico E Stamatakis EA Pérez-García M Trait impulsivity and prefrontal gray matter reductions in cocaine dependent individuals. Drug Alcohol Depend (2012) 125(3):208–14 10.1016/j.drugalcdep.2012.02.012 22391134

[14] O’Malley S Adamse M Heaton RK Gawin FH Neuropsychological impairment in chronic cocaine abusers. Am J Drug Alcohol Abuse (1992) 18(2):131–44 10.3109/00952999208992826 1562011

[15] Gillen RW Kranzler HR Bauer LO Burleson JA Samarel D Morrison DJ Neuropsychologic findings in cocaine-dependent outpatients. Prog Neuropsychopharmacol Biol Psychiatry (1998) 22(7):1061–76 10.1016/S0278-5846(98)00057-8 9829288

[16] Cunha PJ Nicastri S Gomes LP Moino RM Peluso MA Neuropsychological impairments in crack cocaine-dependent inpatients: preliminary findings. Rev Bras Psiquiatr (2004) 26(2):103–6 10.1590/S1516-44462004000200007 15517061

[17] Cunha PJ Nicastri S de Andrade AG Bolla KI The frontal assessment battery (FAB) reveals neurocognitive dysfunction in substance-dependent individuals in distinct executive domains: abstract reasoning, motor programming, and cognitive flexibility. Addict Behav (2010) 35(10):875–81 10.1016/j.addbeh.2010.05.005 20584570

[18] Cunha PJ Bechara A de Andrade AG Nicastri S Decision-making deficits linked to real-life social dysfunction in crack cocaine-dependent individuals. Am J Addict. (2011) 20(1):78–86 10.1111/j.1521-0391.2010.00097.x 21175924 PMC3124808

[19] Vonmoos M Hulka LM Preller KH Jenni D Baumgartner MR Stohler R Cognitive dysfunctions in recreational and dependent cocaine users: role of attention-deficit hyperactivity disorder, craving and early age at onset. Br J Psychiatry (2013) 203(1):35–43 10.1192/bjp.bp.112.118091 23703315

[20] Dubois B Slachevsky A Litvan I Pillon B The FAB: a frontal assessment battery at bedside. Neurology (2000) 55:1621–6 10.1212/WNL.55.11.1621 11113214

[21] Aharonovich E Hasin DS Brooks AC Liu X Bisaga A Nunes EV Cognitive deficits predict low treatment retention in cocaine dependent patients. Drug Alcohol Depend (2006) 81:313–22 10.1016/j.drugalcdep.2005.08.003 16171953

[22] Verdejo-García A Betanzos-Espinosa P Lozano OM Vergara-Moragues E González-Saiz F Fernández-Calderón F Self-regulation and treatment retention in cocaine dependent individuals: a longitudinal study. Drug Alcohol Depend (2012) 122(1–2):142–8 10.1016/j.drugalcdep.2011.09.025 22018602

[23] Aharonovich E Nunes E Hasin D Cognitive impairment, retention and abstinence among cocaine abusers in cognitive-behavioral treatment. Drug Alcohol Depend (2003) 71(2):207–11 10.1016/S0376-8716(03)00092-9 12927659 PMC5804498

[24] Dolan SL Bechara A Nathan PE Executive dysfunction as a risk marker for substance abuse: the role of impulsive personality traits. Behav Sci Law (2008) 26(6):799–822 10.1002/bsl.845 19039793

[25] Glass JM Buu A Adams KM Nigg JT Puttler LI Jester JM Effects of alcoholism severity and smoking on executive neurocognitive function. Addiction (2009) 104(1):38–48 10.1111/j.1360-0443.2008.02415.x 19133887 PMC2734473

[26] Verdejo-García A Bechara A Recknor EC Perez-Garcia M Executive dysfunction in substance dependent individuals during drug use and abstinence: an examination of the behavioral cognitive and emotional correlates of addiction. J Int Neuropsychol Soc (2006) 12:405–15 16903133 10.1017/s1355617706060486

[27] Fillmore MT Rush CR Impaired inhibitory control of behavior in chronic cocaine users. Drug Alcohol Depend (2002) 66(3):265–73 10.1016/S0376-8716(01)00206-X 12062461

[28] Robinson ES Eagle DM Economidou D Theobald DE Mar AC Murphy ER Behavioural characterisation of high impulsivity on the 5-choice serial reaction time task: specific deficits in ‘waiting’ versus ‘stopping’. Behav Brain Res (2009) 196(2):310–6 10.1016/j.bbr.2008.09.021 18940201

[29] Ersche KD Turton AJ Pradhan S Bullmore ET Robbins TW Drug addiction endophenotypes: impulsive versus sensation-seeking personality traits. Biol Psychiatry (2010) 68(8):770–3 10.1016/j.biopsych.2010.06.015 20678754 PMC3485555

[30] Robbins TW Gillan CM Smith DG de Wit S Ersche KD Neurocognitive endophenotypes of impulsivity and compulsivity: towards dimensional psychiatry. Trends Cogn Sci (2012) 16(1):81–91 10.1016/j.tics.2011.11.009 22155014

[31] American Psychiatric Association Diagnostic and Statistical Manual of Mental Disorders, 4th edition – Text Revised. Washington, DC: American Psychiatric Association Press (2000).

[32] Rounsaville BJ Anton SF Carroll K Budde D Prusoff BA Gawin F Psychiatric diagnoses of treatment-seeking cocaine abusers. Arch Gen Psychiatry (1991) 48(1):43–51 10.1001/archpsyc.1991.01810250045005 1984761

[33] Seidman LJ Biederman J Weber W Hatch M Faraone SV Neuropsychological function in adults with attention-deficit hyperactivity disorder. Biol Psychiatry (1998) 44(4):260–8 10.1016/S0006-3223(97)00392-2 9715357

[34] Shallice T Marzocchi GM Coser S Del Savio M Meuter RF Rumiati RI Executive function profile of children with attention deficit hyperactivity disorder. Dev Neuropsychol (2002) 21(1):43–71 10.1207/S15326942DN2101_3 12058835

[35] Tseng WL Gau SS Executive function as a mediator in the link between attention-deficit/hyperactivity disorder and social problems. J Child Psychol Psychiatry (2013) 54(9):996–1004 10.1111/jcpp.12072 23574361

[36] Slaats-Willemse D Swaab-Barneveld H de Sonneville L van der Meulen E Buitelaar J Deficient response inhibition as a cognitive endophenotype of ADHD. J Am Acad Child Adolesc Psychiatry (2003) 42(10):1242–8 10.1097/00004583-200310000-00016 14560175

[37] Horner MD Attentional functioning in abstinent cocaine abusers. Drug Alcohol Depend (1999) 54:19–33 10.1016/S0376-8716(98)00141-0 10101614

[38] Beatty WW Katzung VM Moreland VJ Nixon SJ Neuropsychological performance of recently abstinent alcoholics and cocaine abusers. Drug Alcohol Depend (1995) 37(3):247–53 10.1016/0376-8716(94)01072-S 7796719

[39] Woicik PA Moeller SJ Alia-Klein N Maloney T Lukasik TM Yeliosof O The neuropsychology of cocaine addiction: recent cocaine use masks impairment. Neuropsychopharmacology (2009) 34(5):1112–22 10.1038/npp.2008.60 18496524 PMC2667096

[40] Beck AT Ward CH Mendelson M Mock J Erbaugh J An inventory for measuring depression. Arch Gen Psychiatry (1961) 4:561–71 10.1001/archpsyc.1961.01710120031004 13688369

[41] Spielberger CD Gorsuch RL Lushene RE Manual for the State-Trait Anxiety Inventory. Palo Alto, CA: Consulting Psychologist Press (1970).

[42] Washton AM Cocaine Addiction: Treatment, Recovery and Relapse Prevention. New York, NY: WW Norton & Company (1989).

[43] McLellan AT Kushner H Metzger D Peters R Smith I Grissom G The fifth edition of the addiction severity index. J Subst Abuse Treat (1992) 9:199–213 10.1016/0740-5472(92)90062-S 1334156

[44] Wechsler D Wechsler Intelligence Scale for Adults-Revised (WAIS-R). New York, NY: The Psychological Corporation (1981).

[45] Lezak MD Howieson DB Loring DW Neuropsychological Assessment. 4th ed New York, NY: Oxford University Press (2004).

[46] Stroop JR Studies of interference in serial verbal reaction. J Exp Psychol (1935) 18:643–62 10.1037/h0054651

[47] Heaton RK Chelune GJ Talley JL Kay GG Curtiss G Wisconsin Card Sorting Test (WCST) – Manual Revised and Expanded. Odessa, FL: Psychological Assessment Resources (1993).

[48] Haaland KY Vranes LF Goodwin JS Garry PJ Wisconsin Card Sort Test performance in a healthy elderly population. J Gerontol (1987) 42(3):345–6 10.1093/geronj/42.3.345 3571874

[49] Cunha JA Trentini CM Argimon IL Oliveira MS Werlang BG Prieb PG Teste Wisconsin de Classificação de Cartas. São Paulo: Casa do Psicólogo (2004).

[50] Cunha PJ Novaes MA Neurocognitive assessment in alcohol abuse and dependence: implications for treatment. Rev Bras Psiquiatr (2004) 26(Suppl 1):S23–7 10.1590/S1516-44462004000500007 15729440

[51] Ward MF Wender PH Reimherr FW The Wender Utah Rating Scale: an aid in the retrospective diagnosis of childhood attention deficit hyperactivity disorder. Am J Psychiatry (1993) 150:885–90 8494063 10.1176/ajp.150.6.885

[52] Bolla KI Eldreth DA London ED Kiehl KA Mouratidis M Contoreggi C Orbitofrontal cortex dysfunction in abstinent cocaine abusers performing a decision-making task. Neuroimage (2003) 19(3):1085–94 10.1016/S1053-8119(03)00113-7 12880834 PMC2767245

[53] Goldstein RZ Volkow ND Drug addiction and its underlying neurobiological basis: neuroimaging evidence for the involvement of the frontal cortex. Am J Psychiatry (2002) 159(10):1642–52 10.1176/appi.ajp.159.10.1642 12359667 PMC1201373

[54] Preller KH Ingold N Hulka LM Vonmoos M Jenni D Baumgartner MR Increased sensorimotor gating in recreational and dependent cocaine users is modulated by craving and attention-deficit/hyperactivity disorder symptoms. Biol Psychiatry (2013) 73(3):225–34 10.1016/j.biopsych.2012.08.003 22959126

[55] Volkow ND Fowler JS Addiction, a disease of compulsion and drive: involvement of the orbitofrontal cortex. Cereb Cortex (2000) 10:318–25 10.1093/cercor/10.3.318 10731226

[56] Dalley JW Everitt BJ Robbins TW Impulsivity, compulsivity, and top-down cognitive-control. Neuron (2011) 69(4):680–94 10.1016/j.neuron.2011.01.020 21338879

[57] Moeller SJ Honorio J Tomasi D Parvaz MA Woicik PA Volkow ND Methylphenidate enhances executive function and optimizes prefrontal function in both health and cocaine addiction. Cereb Cortex (2012). 10.1093/cercor/bhs345 23162047 PMC3920764

[58] Volkow ND Wang GJ Tomasi D Kollins SH Wigal TL Newcorn JH Methylphenidate-elicited dopamine increases in ventral striatum are associated with long-term symptom improvement in adults with attention deficit hyperactivity disorder. J Neurosci (2012) 32(3):841–9 10.1523/JNEUROSCI.4461-11.2012 22262882 PMC3350870

[59] Martinotti G Di Nicola M Romanelli R Andreoli S Pozzi G Moroni N High and low dosage oxcarbazepine versus naltrexone for the prevention of relapse in alcohol-dependent patients. Hum Psychopharmacol (2007) 22(3):149–56 10.1002/hup.833 17397097

[60] Thorens G Billieux J Manghi R Khan R Khazaal Y Zullino DF The potential interest of topiramate in addiction. Curr Pharm Des (2011) 17(14):1410–5 10.2174/138161211796150864 21524260

[61] Martinotti G Pregabalin in clinical psychiatry and addiction: pros and cons. Expert Opin Investig Drugs (2012) 21(9):1243–5 10.1517/13543784.2012.703179 22725618

[62] Wilens TE Adamson J Monuteaux MC Faraone SV Schillinger M Westerberg D Effect of prior stimulant treatment for attention-deficit/hyperactivity disorder on subsequent risk for cigarette smoking and alcohol and drug use disorders in adolescents. Arch Pediatr Adolesc Med (2008) 162(10):916–21 10.1001/archpedi.162.10.916 18838643 PMC2760975

[63] Cservenka A Herting MM Nagel BJ Atypical frontal lobe activity during verbal working memory in youth with a family history of alcoholism. Drug Alcohol Depend (2012) 123(1–3):98–104 10.1016/j.drugalcdep.2011.10.021 22088655 PMC3294260

[64] Gierski F Hubsch B Stefaniak N Benzerouk F Cuervo-Lombard C Bera-Potelle C Executive functions in adult offspring of alcohol-dependent probands: toward a cognitive endophenotype? Alcohol Clin Exp Res (2013) 37(Suppl 1):E356–63 10.1111/j.1530-0277.2012.01903.x 23240659

[65] Jupp B Dalley JW Behavioral endophenotypes of drug addiction: etiological insights from neuroimaging studies. Neuropharmacology (2013). 10.1016/j.neuropharm.2013.05.041 23756169

[66] Ersche KD Turton AJ Chamberlain SR Müller U Bullmore ET Robbins TW Cognitive dysfunction and anxious-impulsive personality traits are endophenotypes for drug dependence. Am J Psychiatry (2012) 169(9):926–36 10.1176/appi.ajp.2012.11091421 22952072 PMC3533378

[67] Verdejo-García A Lawrence AJ Clark L Impulsivity as a vulnerability marker for substance-use disorders: review of findings from high-risk research, problem gamblers and genetic association studies. Neurosci Biobehav Rev (2008) 32(4):777–810 10.1016/j.neubiorev.2007.11.003 18295884

[68] Kreek MJ Nielsen DA Butelman ER LaForge KS Genetic influences on impulsivity, risk taking, stress responsivity and vulnerability to drug abuse and addiction. Nat Neurosci (2005) 8(11):1450–7 10.1038/nn1583 16251987

[69] Dias G Mattos P Coutinho G Segenreich D Saboya E Ayrão V Agreement rates between parent and self-report on past ADHD symptoms in an adult clinical sample. J Atten Disord (2008) 12(1):70–5 10.1177/1087054707311221 18192619

